# MDM2 Amplified Sarcomas: A Literature Review

**DOI:** 10.3390/diagnostics11030496

**Published:** 2021-03-11

**Authors:** Raf Sciot

**Affiliations:** Department of Pathology, University Hospital, University of Leuven, 3000 Leuven, Belgium; raf.sciot@uzleuven.be

**Keywords:** MDM2 amplification, well-differentiated liposarcoma/atypical lipomatous tumor, dedifferentiated liposarcoma, intimal sarcoma low grade osteosarcoma

## Abstract

Murine Double Minute Clone 2, located at 12q15, is an oncogene that codes for an oncoprotein of which the association with p53 was discovered 30 years ago. The most important function of MDM2 is to control p53 activity; it is in fact the best documented negative regulator of p53. Mutations of the tumor suppressor gene p53 represent the most frequent genetic change in human cancers. By overexpressing MDM2, cancer cells have another means to block p53. The sarcomas in which MDM2 amplification is a hallmark are well-differentiated liposarcoma/atypical lipomatous tumor, dedifferentiated liposarcoma, intimal sarcoma, and low-grade osteosarcoma. The purpose of this review is to summarize the typical clinical, histopathological, immunohistochemical, and genetic features of these tumors.

## 1. Introduction

*Murine Double Minute Clone 2* is an oncogene, the function of which was first described in DNA associated with paired acentric chromatin bodies, termed double minutes, harbored in spontaneously transformed mouse 3T3 fibroblasts [1]. *MDM2* (located at 12q15) codes for an oncoprotein of which the association with p53 was discovered 30 years ago. The protein functions as an E3 ubiquitin ligase that targets the p53 protein for proteasomal degradation. Thus, the most important function of *MDM2* is to control p53 activity; it is in fact the best documented negative regulator of p53. For normal cells, it is of utmost importance that p53 is maintained at low levels since p53 has potent growth suppressive activities. In fact, p53 is lethal in the absence of *MDM2*. Mouse *MDM2*−/− embryos are inviable and deletion of p53 rescues these embryos. There are different mechanisms by which *MDM2* can block p53 activity. On the one hand, *MDM2* stimulates the degradation of p53 by escorting p53 from the nucleus to the cytoplasm and by catalyzing the ubiquitination of p53 and hence, proteosomal degradation. On the other hand, *MDM2* blocks the activity of intact p53 by inhibiting the p53 transactivation domain. Indeed, it is well known that p53 trans-activates a number of genes that mediate cell cycle arrest or apoptosis [1]. The oncogenic activities of *MDM2* extend beyond the regulation of p53. Thus, MDM2-mediated ubiquitination of various transcription factors (ATF, E2F and others), of the retinoblastoma protein, and the histones H2A and H2B can influence cell proliferation, DNA repair, transcription, ribosome biosynthesis and cell fate. In addition, chromatin-bound *MDM2* plays a key role in serine metabolism [2].

Mutations of the tumor suppressor gene p53 represent the most frequent genetic change in human cancers. By overexpressing *MDM2*, cancer cells have another means to block p53. It is no surprise that tumors that show *MDM2* amplification do not require p53 mutations.

The overall *MDM2* gene amplification frequency in human cancer varies between 3.5 and 4.4% [3,4]. Some gliomas, carcinomas and hematological malignancies have been described to amplify *MDM2*, but this feature is most frequently seen in sarcomas, the topic of the review.

The sarcomas in which *MDM2* amplification is a hallmark are well-differentiated liposarcoma/atypical lipomatous tumor, dedifferentiated liposarcoma, intimal sarcoma, and low-grade osteosarcoma. Myxofibrosarcomas, malignant peripheral nerve sheath tumors and undifferentiated sarcomas can occasionally show *MDM2* amplification as well. Very recently it was described that a rare subtype of endometrial sarcoma, characterized by *BCOR* rearrangement, also seems to carry *MDM2* amplifications [5]. 

*MDM2* has become a hot topic in cancer treatment, because anti-*MDM2* therapy has become a reality: we actually know of molecules that block the *MDM2*-p53 interaction, and thus, reestablish wild type p53 activity [6,7,8,9]. First-generation small molecule inhibitors of *MDM2*, nutlins, were identified in 2004. Nutlin-3A, a pharmacological inhibitor of the *MDM2*–p53 interaction, stabilizes p53; however, its clinical efficacy is very low and severe thrombocytopenia represents a dose-limiting toxicity. Recent novel second-generation molecules that interfere with *MDM2*, or the *MDM2*–p53 interaction, seem to be more promising [2,10,11,12]. Interestingly, *MDM2* is also used as a target in many non-neoplastic diseases, which underscores its role beyond oncology [13].

## 2. Well-Differentiated Liposarcoma/Atypical Lipomatous Tumor (WDL/ATL)

**Figure 1:**
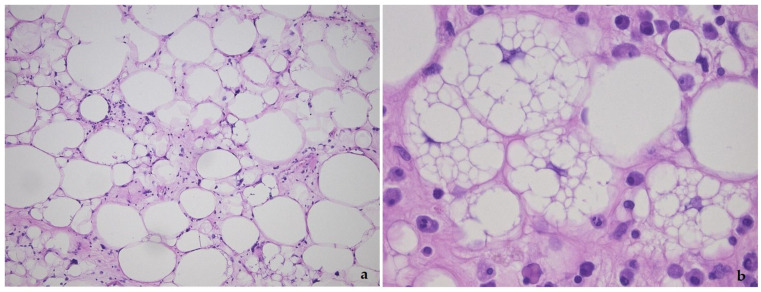
Lipoma-like variant of well-differentiated liposarcoma/atypical lipomatous tumor, showing the variation in adipocyte size, hyperchromatic nuclei and lipoblasts (**a**), detail of lipoblasts with the typical punched-out nucleus in (**b**).

**Figure 2:**
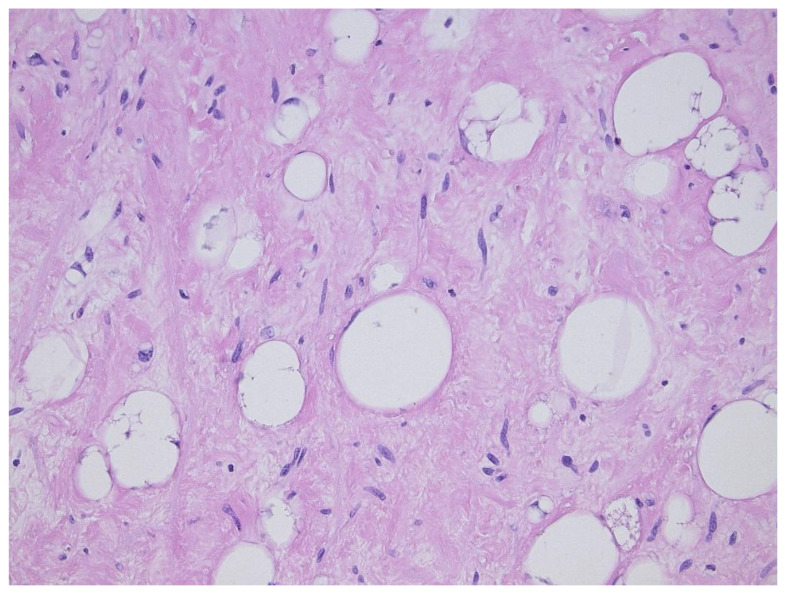
Sclerosing variant of well-differentiated liposarcoma/atypical lipomatous tumor, highlighting the scattered lipoblasts in a sclerotic background.

**Figure 3:**
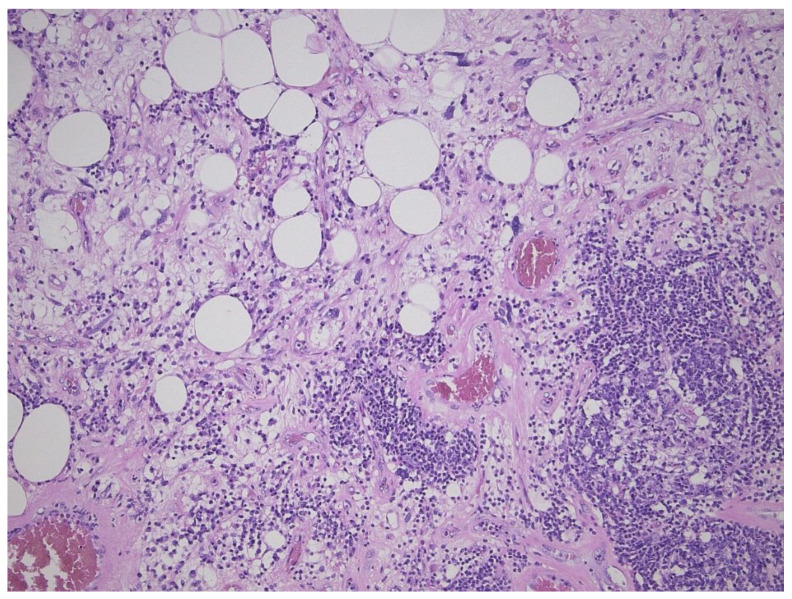
Inflammatory variant of well-differentiated liposarcoma/atypical lipomatous tumor, dominated by a prominent lymphoplasmocytic infiltration. Note the atypical fat cells as well.

**Figure 4:**
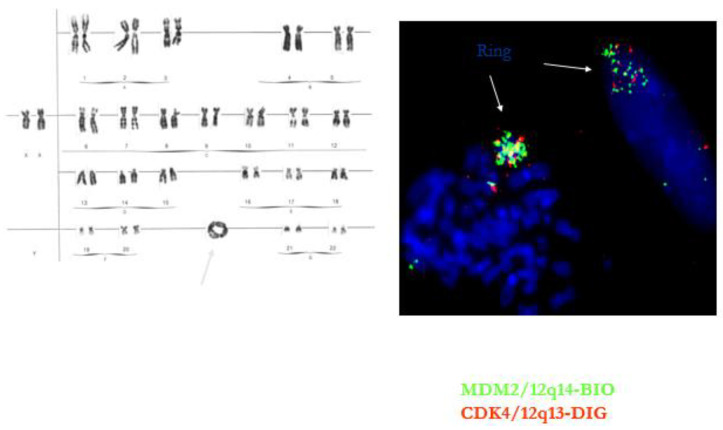
Ring chromosome in well-differentiated liposarcoma/atypical lipomatous tumor. The ring contains multiple copies of the MDM2 and CDK4 gene.

**Figure 5:**
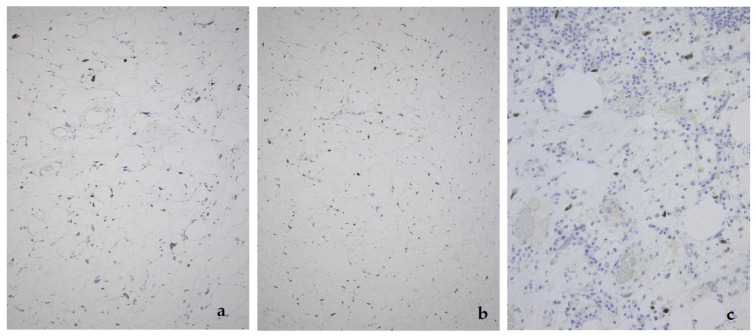
MDM2 expression in the lipoma-like (**a**), sclerosing (**b**), and inflammatory variant (**c**) of well-differentiated liposarcoma/atypical lipomatous tumor.

This tumor represents the most common malignant adipocytic neoplasm and is mainly seen in middle-aged adults. The deep soft tissues of the extremities are most frequently involved (75%), followed by the retroperitoneum (20%). The groin, paratesticulum and mediastinum are also known locations. The overall mortality is zero for lesions of the extremities and more than 80% for exactly the same tumors in the retroperitoneum. Therefore, the term “atypical lipomatous tumor” is used for lesions in surgically amenable sites where excision can be curative, like the extremities, and “well differentiated liposarcoma” for tumors presenting in the retroperitoneum, spermatic cord and mediastinum, where complete removal is often not possible leading to high morbidity and mortality. Three morphological subtypes are classically described: lipoma-like, sclerosing and inflammatory [14]. In the lipoma-like variant, the basic morphological hallmark is the presence of variation in adipocyte diameter, hyperchromatic/atypical nuclei and lipoblasts (Figure 1). In the sclerosing variant, scattered bizarre hyperchromatic cells and lipoblasts are seen in an extensive fibrillary stroma, varying from dens to myxoid (Figure 2). This subtype is most often seen in the retroperitoneum or paratesticular area. In the inflammatory variant, mainly seen in the retroperitoneum, a lymphoplasmocytic inflammatory infiltrate predominates and obscures the adipocytic component, to the extent that it can mimic inflammatory myofibroblastic tumor, Castleman’s disease or even a lymphoma (Figure 3). These phenotypic variants can be admixed in the same tumor. Whatever it looks like, WDL/ATL is characterized by giant marker and/or supernumerary ring chromosomes, both of which contain multiple copies of *MDM2*. This amplification results in nuclear *MDM2* protein overexpression. There is frequently co-amplification of other genes of the 12q14-15 region, like *CDK4, GLI1* and *HMGA2*, but *MDM2* amplification is the main driver (Figure 4). Immunohistochemistry and/or FISH for *MDM2* is frequently used to support the diagnosis of WDL/ATL, FISH being much more sensitive and specific than protein detection (Figure 5) [15,16]. *MDM2* RNA ISH seems to be as performant as DNA FISH [17]. An important pitfall is the presence of nuclear *MDM2* immunoreactivity in histiocytes/lipophages which are very often seen in traumatized fatty tumors. In addition, any cytoplasmic positivity is not diagnostic. The pathologist is often faced with lesions in which the histological changes are not convincing enough for WDL/ATL and thus, the differential diagnosis with an ordinary lipoma cannot be made on morphological grounds alone. This does not mean that every lipoma should be FISHed for *MDM2*, the following features justify the use of this test: (1) recurrent lesion, (2) deep extremity lesion larger than 10 cm in a patient over 50 years of age, (3) lesion with equivocal atypia, (4) lesion in the retroperitoneum/pelvis/abdomen, and (5) lesions not fitting the above criteria but having worrisome clinical or radiological features [18].

A lack of *MDM2* amplification does not entirely preclude the diagnosis of ALT/WDL. In Li–Fraumeni syndrome, the occurrence of p53+/*MDM2*− ALT/WDL can be an early expression of the disease [19].

It is of interest that in the past, a well-differentiated spindle cell liposarcoma subtype was described. This group of tumors is now called atypical spindle cell lipoma, since it has a different genetic background, relating it to spindle cell lipoma, with deletion of the *Rb-1* gene [20,21]. 

Very recently, rare cases of “dysplastic lipoma”, “anisometric cell lipoma”, or “minimally ALT” have been reported. These tumors, which do not fit neatly into the category of lipoma or ALT, show prominent variation in adipocyte size, frequent single-cell adipocytic necrosis, limited nuclear enlargement and hyperchromasia, and minimally thickened fibrous septa. These tumors do not show *MDM2* amplification as determined by FISH, but are positive for *MDM2* RNA-ISH. In addition, they can show deletion of the *RB-1* gene on 13q14 [22,23,24]. Clinically, local recurrence in dysplastic lipoma is estimated to be approximately 10%, which is intermediate between lipoma and ALT. Further studies are warranted to clarify the exact classification of these lesions.

## 3. Dedifferentiated Liposarcoma

**Figure 6:**
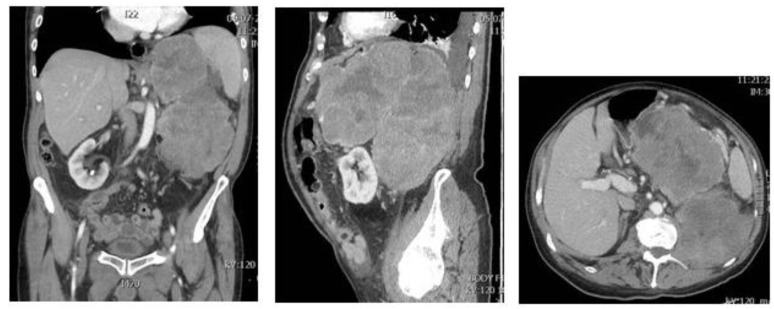
MRI pictures of a big retroperitoneal tumor in an 82-years-old male patient.

**Figure 7:**
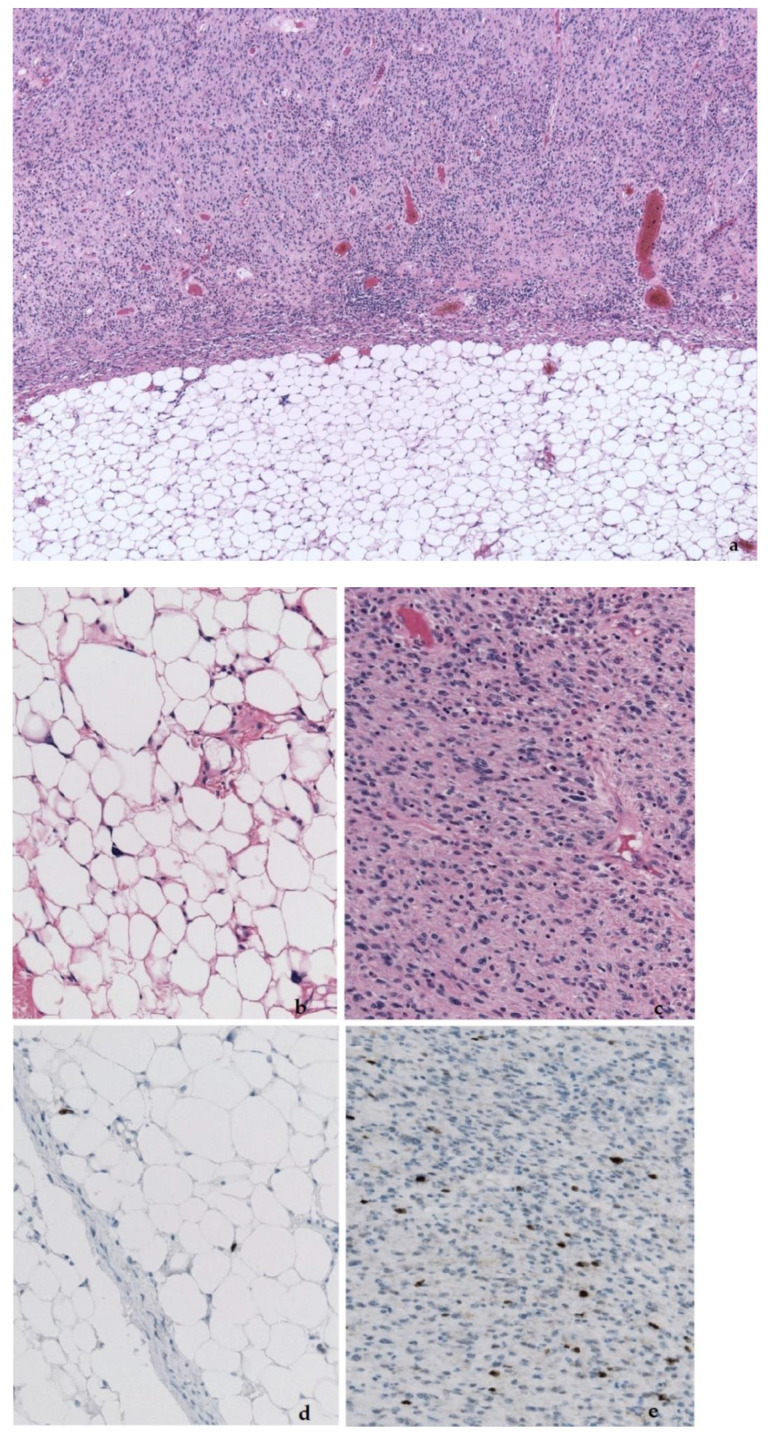
Abrupt transition of the well-differentiated lipoma-like liposarcoma part to the non-lipogenic sarcomatous part (**a**). Detail of both parts (**b**,**c**), and *MDM2* expression in both parts (**d**,**e**).

**Figure 8:**
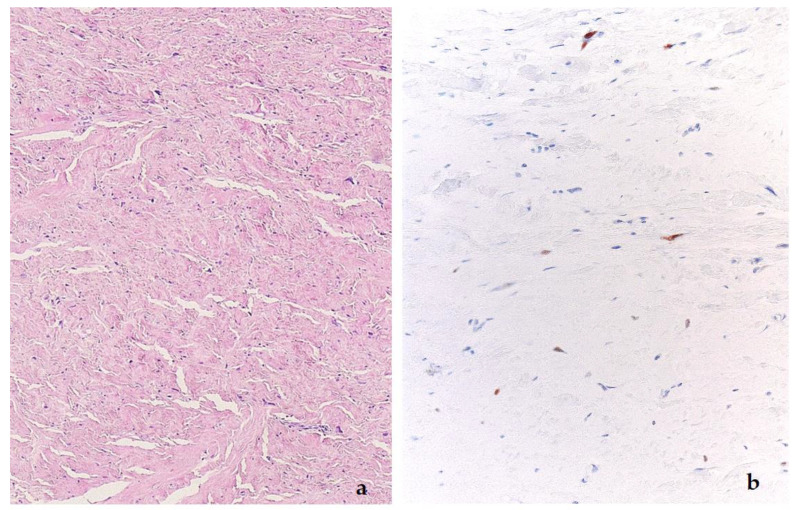
Scar-like phenotype of a dedifferentiated liposarcoma, H&E (**a**), and *MDM2* stain (**b**).

**Figure 9:**
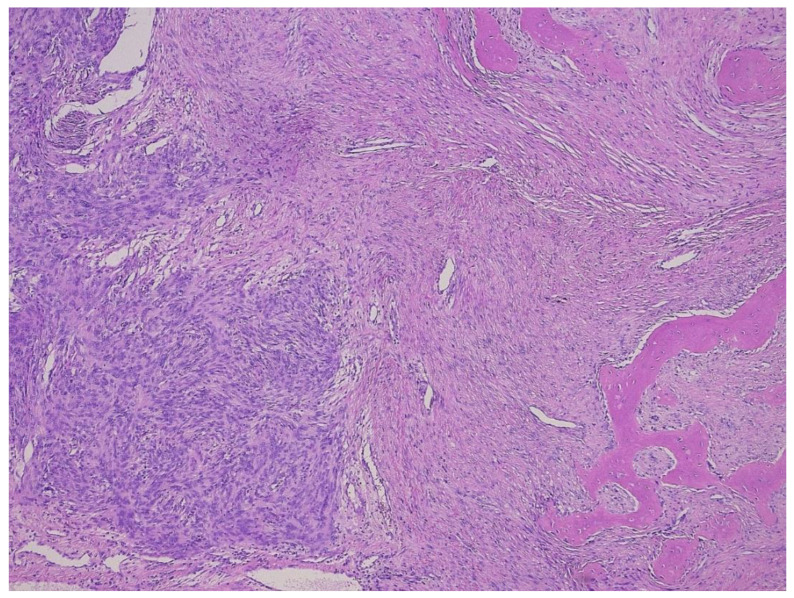
Meningothelial-like whorls and metaplastic bone formation in a retroperitoneal dedifferentiated liposarcoma (H&E).

Dedifferentiated liposarcoma (DDL) is defined as the transition of WDL/ATL towards nonlipogenic sarcoma, either in the primary tumor or in a recurrence. The well-differentiated component can be absent. A total of 90% of DDL present de novo, +/− 10% of WDL/ATL dedifferentiate into DDL. Patients are most often older than 45 years. The retroperitoneum is the most frequent site (80%), and DDL is the most frequent retroperitoneal sarcoma (Figure 6). Other sites are the deep soft tissue of the extremities, the funiculus spermaticus/paratesticulum and mediastinum. The transition to the nonlipogenic sarcoma is usually abrupt and the latter most often looks like undifferentiated pleomorphic or spindle cell sarcoma, but myxoid sarcoma-like pictures, or low grade desmoid-like areas, can be seen as well (Figure 7 and Figure 8). Heterologous differentiation is present in up to 10%, myogenic (leiomyo-, or rhabdomyosarcoma) being most frequent, followed by osteo- or chondrosarcoma-like phenotypes [25,26]. A peculiar type is the DDL with meningothelial-like whorls and metaplastic bone formation, typically seen in the retroperitoneum (Figure 9) [27]. In the past, an inflammatory type of malignant fibrous histiocytoma, rich in neutrophils and also occurring in the retroperitoneum, was described. This tumor represents another phenotype of DDL [26]. In fact, DDL can mimic any type of sarcoma and *MDM2* immunohistochemistry/FISH should be part of the panel of tests in any sarcoma occurring at any site, certainly if it is undifferentiated and pleomorphic. Thus, if one is confronted with an undifferentiated pleomorphic or spindle cell sarcoma, even if there is virtually no fat, the finding of MDM2 expression/amplification will indicate that it concerns a DDL [28]. An interesting but unusual form of dedifferentiation is the so-called homologous lipogenic dedifferentiation, which is characterized by big atypical lipoblasts, exactly as seen in pleomorphic liposarcoma [29]. MDM2 expression usually is very prominent in the dedifferentiated area as opposed to the well-differentiated part (Figure 7). Once dedifferentiation is present, the tumor gains metastatic potential but is less aggressive than other pleomorphic sarcomas. Recurrence is seen in at least 40% of cases, metastatic rates vary between 15 and 30% and tumor-related death is +/− 28% at 5-year follow-up. This figure is without any doubt much higher at 10 to 20 years [25]. Recent studies indicate that high grade dedifferentiation and the presence of myogenic (particularly rhabdomyoblastic) dedifferentiation correlate with a worse prognosis [30,31,32]. As in WDL/ATL, complete surgical resection is the mainstay of treatment. DDL also harbors high level amplifications of 12q14-15, including the *MDM2* and *CDK4* genes. The degree of MDM2 amplification might affect clinical outcome, although prospective studies are needed to objectivate this finding [32]. As opposed to WDL/ATL, DDL shows other genetic changes, as 6q23 and 1p32 co-amplifications. In this respect, *JUN* (1p32.2) is of interest since its amplification is probably involved in the progression from WDL/ATL to DDL. Another feature that favors dedifferentiation might be the loss of *HMGA2* overexpression, which is frequent in DDL [33]. A recent study on the integrated exome and RNA sequencing of 115 DDLs showed elegantly that gains of several somatic copy-number alternations and chromosomal rearrangements, including the *DNM3OS*-fusion genes and the loss of *G0S2* and *DGAT2*, contribute to the cell cycle progression and impairment of adipogenesis [34].

## 4. Intimal Sarcoma

**Figure 10:**
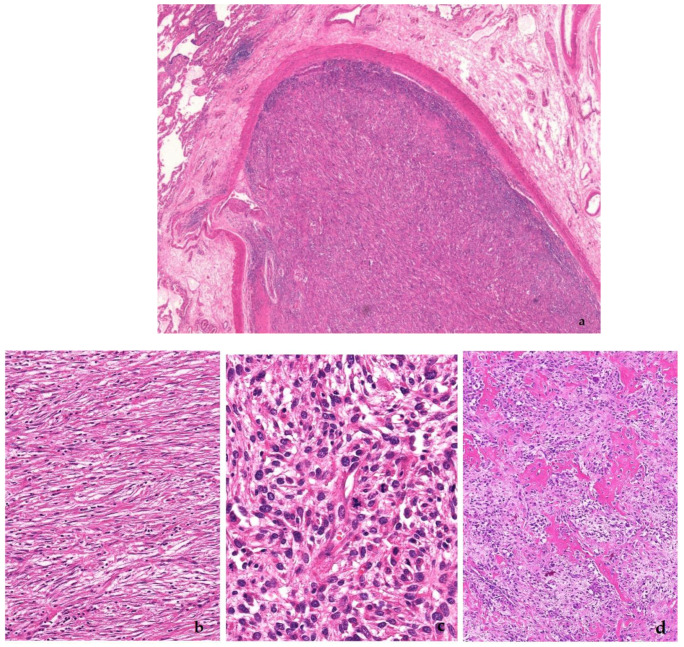

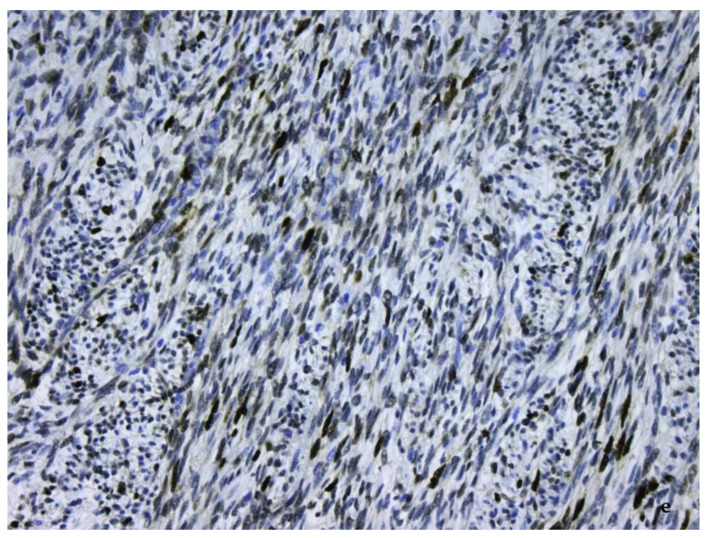
Intimal sarcoma. Note the intravascular location (**a**), and the heterogeneous outlook of the tumor, ranging from bland spindled (**b**), to pleomorphic (**c**), to osteosarcoma-like (**d**). *MDM2* expression is seen in (**e**).

Intimal sarcomas are very rare tumors that develop in the wall of large blood vessels, the proximal pulmonary arteries being the most frequent location [35]. This explains that the clinical presentation often relates to pulmonary embolic disease, due to intravascular tumoral spread. The aorta is the main involved vessel of the peripheral circulation, and intimal sarcoma has recently been described to be the most frequent primary cardiac sarcoma [36]. On histology, these tumors present as intraluminal lesions and can have a very heterogeneous, non-distinctive phenotype. The picture ranges from a bland spindle cell proliferation to a very anaplastic and pleomorphic type of undifferentiated sarcoma. The matrix can be collagenous, myxoid, or can even show lace-like osteoid or chondroid differentiation (Figure 10). Rarely the picture can mimic a leiomyo-, rhabdomy-, or angiosarcoma. There is no specific immunohistochemical marker, but *EGFR* and nuclear *MDM2* expression are seen in +/− 70% of cases (Figure 10). Amplification of the 12q12-15 region, including *CDK4* and *MDM2* is a hallmark and is often accompanied by amplification and activation of *platelet-derived growth factor alpha* (at 4q12) and *epidermal growth factor receptor* (at 7p11) [35,37]. The prognosis is very poor, which relates to the embolic dissemination. Lung metastasis is seen in about 40% of patients, and extrathoracic spread in +/− 20%, including the brain, skin and lymph nodes. Survival is rare beyond 3 years [38,39].

## 5. Low-Grade Osteosarcoma

**Figure 11:**
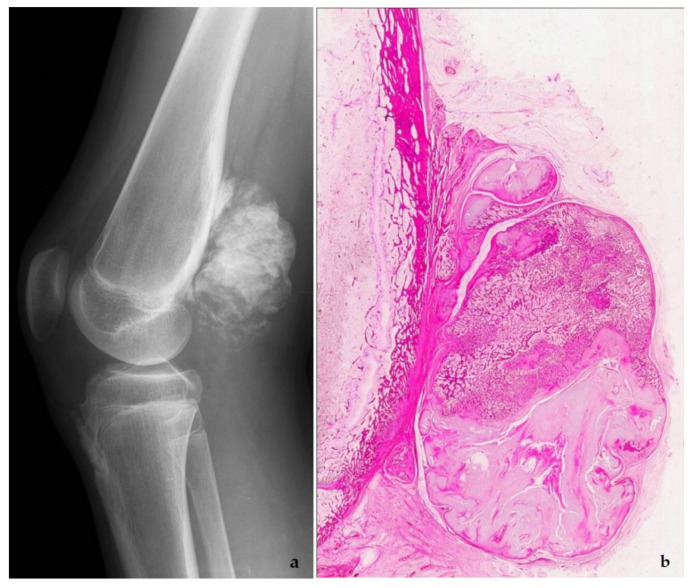

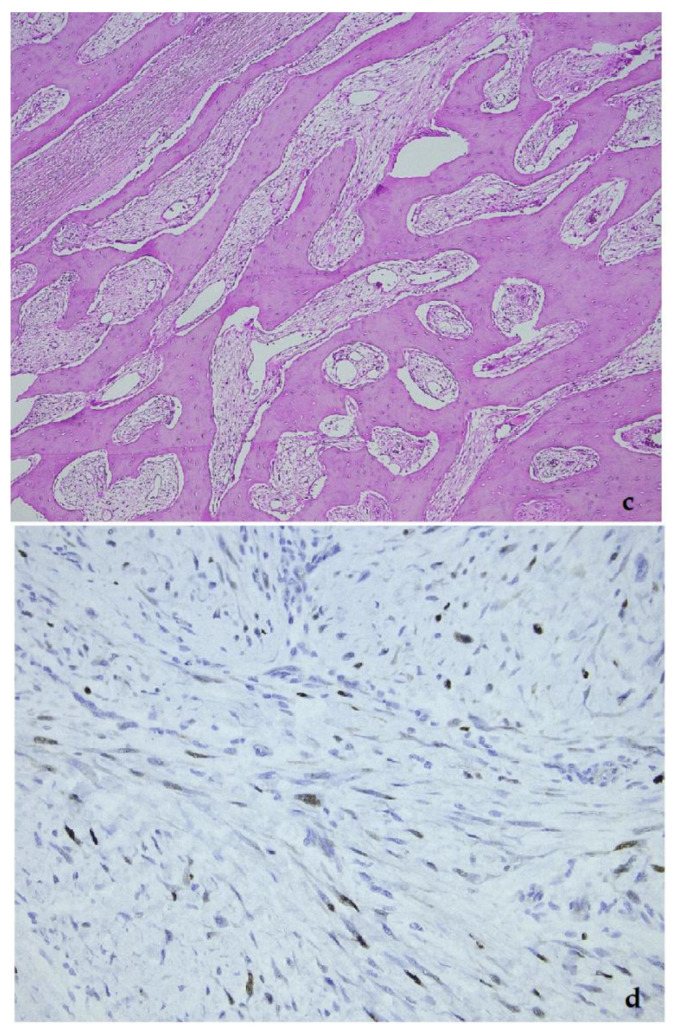
Parosteal osteosarcoma, typically presenting as a mineralized mass at the back of the distal femur, X-ray (**a**), and whole mount section (**b**). At high power, the tumor consists of parallel bone trabeculae and a typical spindle cell proliferation (**c**). *MDM2* expression is present as well (**d**).

**Figure 12:**
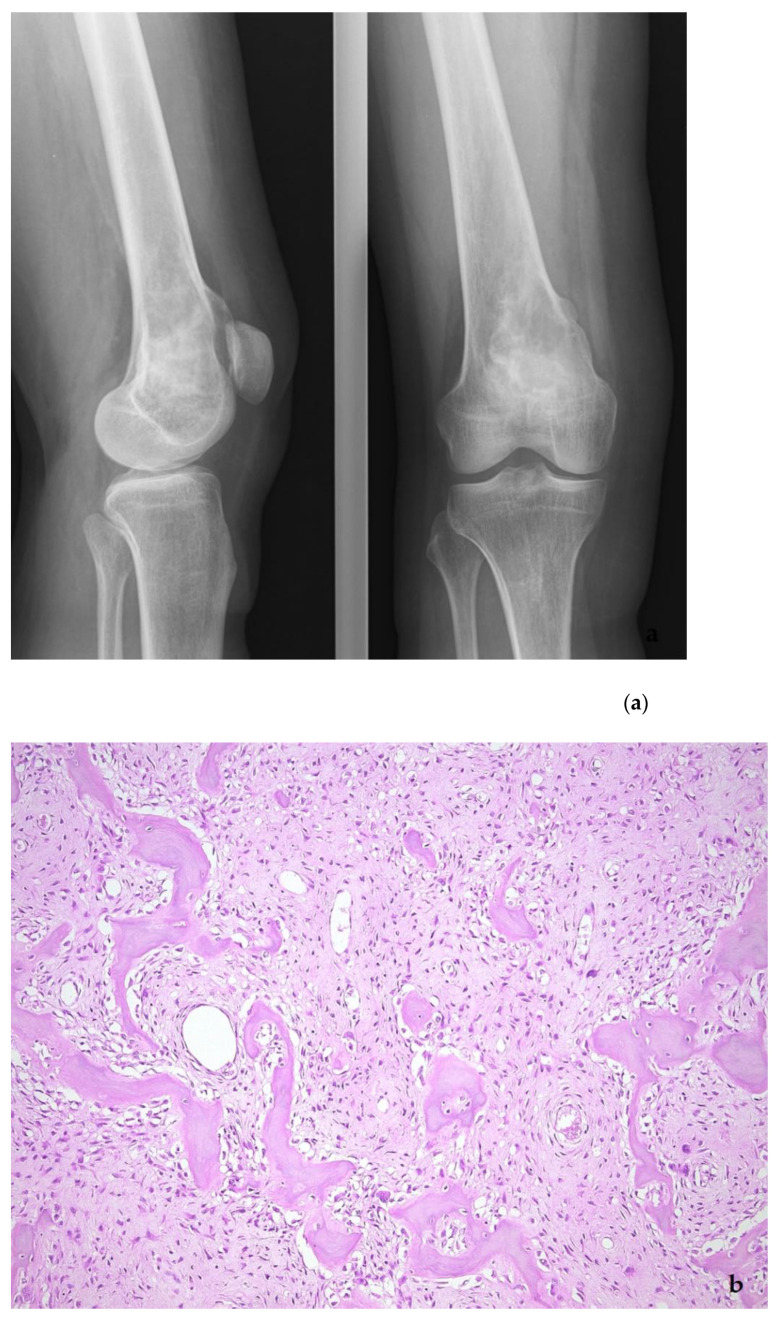

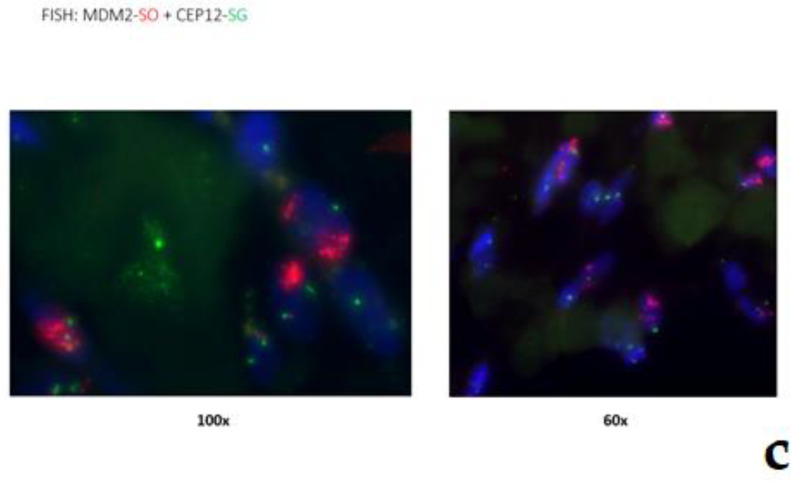
Low-grade central osteosarcoma, showing a lytic lesion the distal femur on X-ray (**a**). On histology, the tumor strongly mimics fibrous dysplasia, with the very irregular bone trabeculae embedded in e fibrous stroma (**b**). The amplification of the *MDM2* gene supports the diagnosis (**c**).

Low-grade osteosarcomas are rare and are subdivided in parosteal and low-grade central osteosarcoma. In both tumors, the diagnosis is often impossible in the absence of imaging features, because they often look deceptively bland. Parosteal osteosarcoma represents 4–5% of all osteosarcomas and is the most frequent surface osteosarcoma. It typically develops on the posterior surface of the distal femur of young adults, mainly in the third decade. The humerus can also be rarely involved. On imaging, a heavily mineralized mass attached to the cortex is seen (Figure 11). Histologically, fascicles of non-atypical spindle cells admixed with parallel bone trabecula are typical (Figure 11). In half of the cases, cartilage islands can be seen, and about half of the tumors transgress the cortex and invade the medullary cavity. Fifteen to 43% dedifferentiate into a high grade (osteo)sarcoma [40]. Low grade central osteosarcoma only accounts for 1–2% of osteosarcomas and is mainly seen in the metaphysis of long bones, tibia and femur being most affected. This tumor rarely involves jaw bones, axial and small tubular bones. Half of the cases occur in the second to third decade. On imaging, a large lytic to mineralized mass is seen, often associated with focal cortical disruption, which can be hard to see (Figure 12). On histology, the tumor consists of a fascicular and moderately cellular fibroblastic proliferation, with minimal or no atypia. The neoplastic bone can present as curved bone trabeculae, mimicking fibrous dysplasia, or as longitudinal seams of bone, as seen in parosteal osteosarcoma (Figure 12). Bone formation can even be absent, resulting in a picture that resembles desmoplastic fibroma or low-grade fibrosarcoma. Very rarely dedifferentiation can occur resulting in a classical high-grade osteosarcoma-like picture [41]. Both tumors harbor long marker or ring chromosomes with amplified 12q13-15 regions, including the *MDM2* and *CDK4* genes [42,43]. In a high proportion of cases, immunohistochemical *MDM2* and/or *CDK4* expression can be found as well. In this respect, search for *MDM2* amplification/expression can be very useful to distinguish these low-grade sarcomas from their—often benign—mimics (Figure 11 and Figure 12) [44]. The prognosis of both neoplasms is excellent, with 5-year survival rates of 90% upon complete resection. Chemotherapy is only administered in cases of dedifferentiation, when the prognosis is that of a conventional osteosarcoma. Interestingly, when low-grade central or parosteal osteosarcoma dedifferentiate into a high-grade sarcoma, they retain their *MDM2*/*CDK4* amplification and overexpression. In addition, the finding of *MDM2* and/or *CDK4* amplification/overexpression in a high-grade osteosarcoma indicates progression/dedifferentiation from a low-grade osteosarcoma and practically excludes a primary conventional high-grade osteosarcoma [43].

In conclusion, the story of *MDM2* in sarcomas gives us insight into ‘*M*’olecular mechanisms. It has created a ‘*D*’iagnostic tool, FISH being more sensitive than immunohistochemistry. *MDM2*-amplified sarcomas ‘*M*’ay be druggable in the future. Finally, it helps us ‘*TO*’ sort out part of the numerous and complex sarcomas.
